# An unusual case of isolated serum γ-glutamyl transferase elevation: slight abnormality also should not be taken lightly

**DOI:** 10.1055/a-2058-8681

**Published:** 2023-05-10

**Authors:** Ke Chen, Yi Lu, Jianyu Yang, Yongwei Sun, Xiujiang Yang

**Affiliations:** 1 Department of Endoscopy, Fudan University Shanghai Cancer Center, Shanghai, China; 2 Department of Oncology, Shanghai Medical College, Fudan University, Shanghai, China; 3 Department of Gastrointestinal Endoscopy, The Sixth Affiliated Hospital, Sun Yat-sen University, Guangzhou, China; 4 Guangdong Provincial Key Laboratory of Colorectal and Pelvic Floor Diseases, The Sixth Affiliated Hospital, Sun Yat-sen University, Guangzhou, China; 5 Department of Biliary-Pancreatic Surgery, and Department of Oncology, State Key Laboratory for Oncogenes and Related Genes, Ren Ji Hospital, School of Medicine, Shanghai Jiao Tong University, Shanghai, China

A 75-year-old man was admitted to our hospital for isolated serum γ-glutamyl transpeptidase (GGT, 147 U/L) elevation, with fluctuation in the past 2 years ( Fig. 1 ). His alanine aminotransferase, glutamic transaminase, bilirubin level, and tumor markers were all in normal range. He had no symptoms, except for mild soreness in the right waist for 2 months. Magnetic resonance imaging (MRI) revealed a low signal in T2 W with dilation of the common bile duct (CBD) to 12 mm and enlargement of the gallbladder ( Fig. 2 ). Hence, endoscopic ultrasound (EUS) was performed for a definite diagnosis. The major papilla was small with a normal surface ( Fig. 3 ). In the distal CBD, a hypoechoic nodule (13.1 × 7.2 mm) was found wriggling along with the sphincter contraction ( Fig. 4 a ). Contrast-enhanced EUS showed the enhancement of the lesion, but the degree was weaker than the peripheral pancreas ( Fig. 4 b , Video 1 ).

**Fig. 1:**
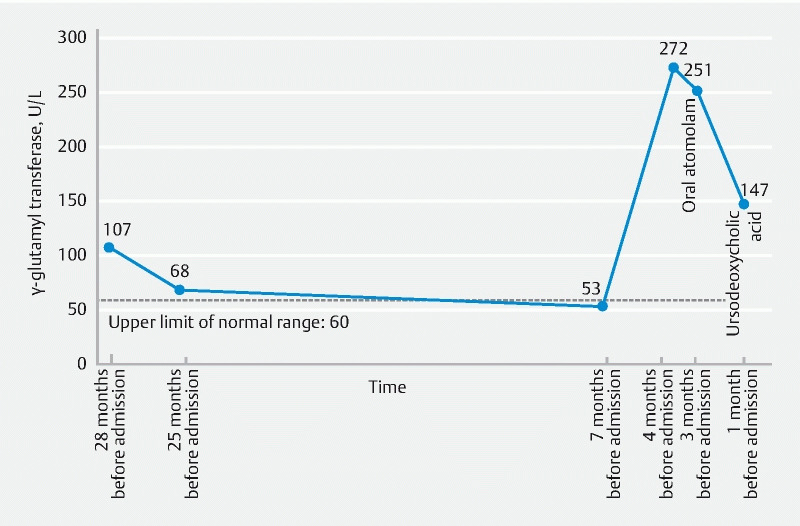
The patientʼs serum γ-glutamyl transpeptidase fluctuation line in the past 2 years.

**Fig. 2:**
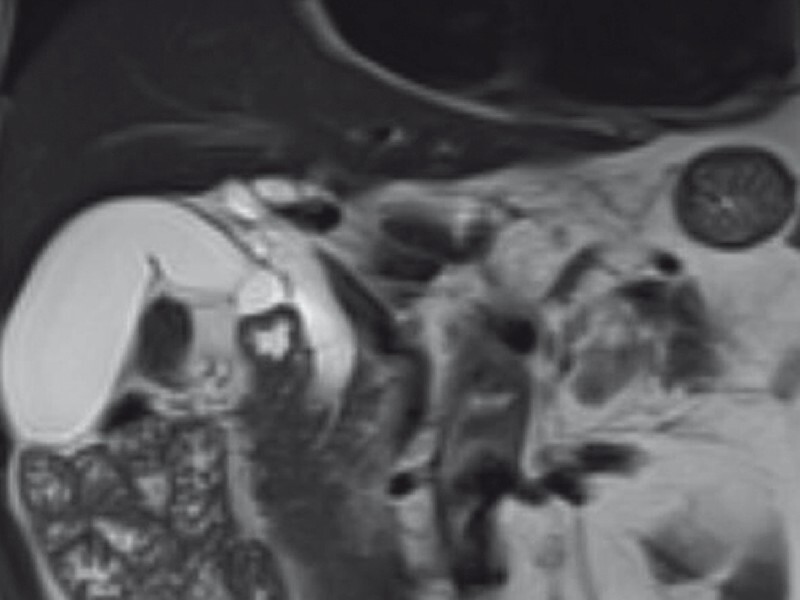
Magnetic resonance imaging revealed a low signal in T2 W with dilation of common bile duct to 12 mm and enlargement of gallbladder.

**Fig. 3:**
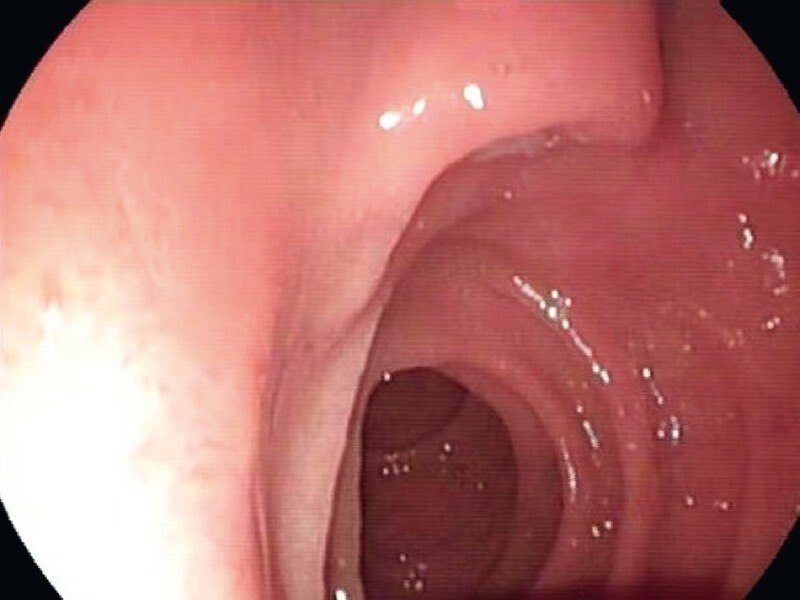
Endoscopy view showed that major papilla was small with normal surface.

**Fig. 4:**
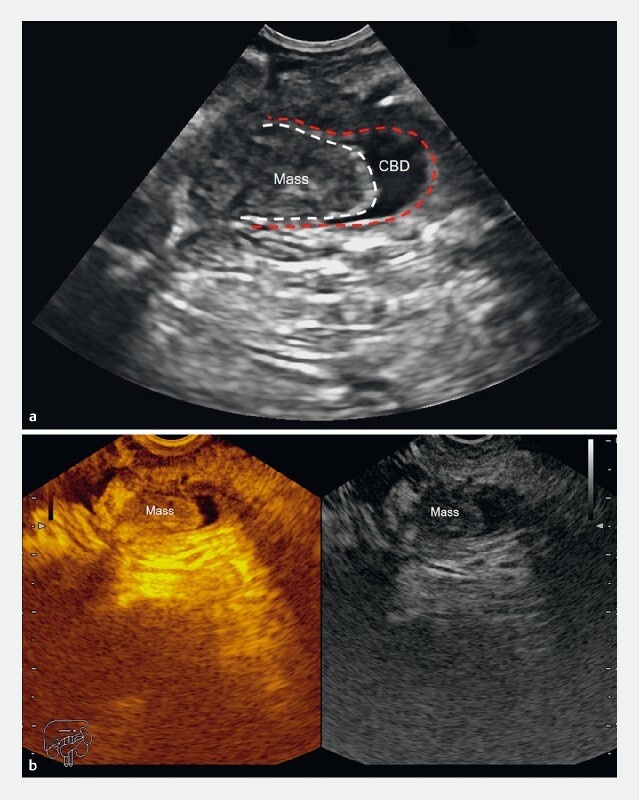
Endoscopic ultrasound images. **a** A hypoechoic nodule in the terminal portion of common bile duct. **b** Contrast-enhanced endoscopic ultrasound showed enhancement of the lesion.

**Video 1** Endoscopic ultrasound showed a hypoechoic nodule in the common bile duct. Contrast-enhanced endoscopic ultrasound showed the enhancement of the lesion, but the degree was weaker than the peripheral pancreas.


The patient was delivered for surgery, and intraoperative choledochoscopy indicated a villous tumor located in the terminal portion of the CBD with a soft texture. Finally, histological diagnosis indicated adenocarcinoma (protruded type, grade II) ( Fig. 5 ). The current case demonstrates a dilated CBD with an isolated serum GGT elevation should be checked for a suspected bile duct neoplasm, even without jaundice. An elevated serum GGT is a marker of hepatobiliary system injury and is associated with a risk of chronic disease 1 and is reported to be independently associated with a risk of cancer 2 and cancer prognosis 3 . Attention should also be paid to a slightly abnormal test; even if there is downward trend, it cannot be taken lightly. Clinically, such a manifestation is prone to be overlooked, especially when a significant mass is obsolete on imaging examination. In this case, the soft nodule restricted excretion of bile flow from the papilla, which verified by the appearance of atrophic papilla. Hence, the elevated pressure in the CBD only led to an elevated GGT, without an abnormal bilirubin level.

**Fig. 5:**
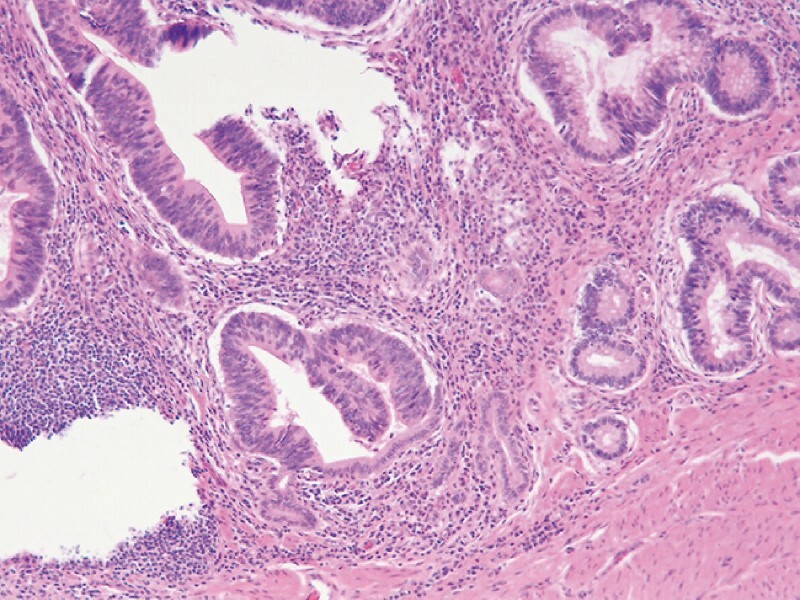
Histological diagnosis indicated adenocarcinoma.

Endoscopy_UCTN_Code_CCL_1AZ_2AC

## Notes

Endoscopy E-Videos

https://eref.thieme.de/e-videos
*E-Videos* is an open access online section of the journal *Endoscopy* , reporting on interesting cases and new techniques in gastroenterological endoscopy. All papers include a high-quality video and are published with a Creative Commons CC-BY license. Endoscopy E-Videos qualify for HINARI discounts and waivers and eligibility is automatically checked during the submission process. We grant 100% waivers to articles whose corresponding authors are based in Group A countries and 50% waivers to those who are based in Group B countries as classified by Research4Life (see: https://www.research4life.org/access/eligibility/ ). This section has its own submission website at https://mc.manuscriptcentral.com/e-videos
